# Cnbp ameliorates Treacher Collins Syndrome craniofacial anomalies through a pathway that involves redox-responsive genes

**DOI:** 10.1038/cddis.2016.299

**Published:** 2016-10-06

**Authors:** Mauro S Porcel de Peralta, Valeria S Mouguelar, María Antonella Sdrigotti, Felipe A A Ishiy, Roberto D Fanganiello, Maria R Passos-Bueno, Gabriela Coux, Nora B Calcaterra

**Affiliations:** 1 IBR (Instituto de Biología Molecular y Celular de Rosario), Consejo Nacional de Investigaciones Científicas y Técnicas (CONICET), Facultad de Ciencias Bioquímicas y Farmacéuticas, Universidad Nacional de Rosario (UNR), Ocampo y Esmeralda, Rosario, 2000 Argentina; 2 Departmento de Genetica e Biologia Evolutiva, Instituto de Biociencias, Universidade de São Paulo, Rua do Matao, 277, Sala 200. Sao Paulo, Brazil

## Abstract

Treacher Collins Syndrome (TCS) is a rare congenital disease (1:50 000 live births) characterized by craniofacial defects, including hypoplasia of facial bones, cleft palate and palpebral fissures. Over 90% of the cases are due to mutations in the *TCOF1* gene, which codifies the nucleolar protein Treacle. Here we report a novel TCS-like zebrafish model displaying features that fully recapitulate the spectrum of craniofacial abnormalities observed in patients. As it was reported for a *Tcof1*^+/−^ mouse model, Treacle depletion in zebrafish caused reduced rRNA transcription, stabilization of Tp53 and increased cell death in the cephalic region. An increase of ROS along with the overexpression of redox-responsive genes was detected; furthermore, treatment with antioxidants ameliorated the phenotypic defects of craniofacial anomalies in TCS-like larvae. On the other hand, Treacle depletion led to a lowering in the abundance of Cnbp, a protein required for proper craniofacial development. *Tcof1* knockdown in transgenic zebrafish overexpressing *cnbp* resulted in barely affected craniofacial cartilage development, reinforcing the notion that Cnbp has a role in the pathogenesis of TCS. The *cnbp* overexpression rescued the TCS phenotype in a dose-dependent manner by a ROS-cytoprotective action that prevented the redox-responsive genes' upregulation but did not normalize the synthesis of rRNAs. Finally, a positive correlation between the expression of *CNBP* and *TCOF1* in mesenchymal cells from both control and TCS subjects was found. Based on this, we suggest CNBP as an additional target for new alternative therapeutic treatments to reduce craniofacial defects not only in TCS but also in other neurocristopathies.

Treacher Collins Syndrome (TCS) (Online Mendelian Inheritance in Man database accession no. 154500) is a congenital craniofacial disorder being characterized by numerous anomalies, restricted primarily to the head and neck. The phenotype of TCS includes hypoplasia of the facial bones, particularly the zygomatic complex and mandible, cleft palate and middle and external ear defects that result in conductive deafness.^1^ Many of the tissues affected in the syndrome arise from the first (maxillary and mandibular) and second (hyoid) pharyngeal arches.^1^ These arches are strongly colonized by cranial neural crest cells (CNCCs), which are migratory multipotent progenitor cells derived from the neuroepithelium. They ultimately form much of the cartilage, bone and connective tissues of the head and the face.^2^

TCS is caused primarily (78–93% of the cases) by autosomal-dominant mutations in the *TCOF1* gene.^3^ The *TCOF1-*coded protein Treacle^4^ participates in ribosomal RNA (rRNA) synthesis via direct binding of upstream binding factor and RNA polymerase I^5^ and interacts with components of the pre-rRNA processing complex^6, 7^ in the nucleolus. A *Tcof1*^+/−^ mouse model was enabled to determine that Treacle is essential for proper development of CNCC by having a key role in ribosome biogenesis.^3^ As a deficiency in ribosome biogenesis may cause stress-induced activation of the tumor protein 53 (Tp53),^8, 9^ it was suggested that the enhanced apoptosis of neuroepithelial cells was due to the activation of Tp53-responsive pro-apoptotic genes.^10^ Supporting this hypothesis, inactivation of Tp53 ameliorates the phenotypic defects showed by *Tcof1*^+/−^ mice. This amelioration occurs apart from the deficits shown in ribosome biogenesis,^10^ suggesting that, in addition to its function in rRNA biosynthesis, Treacle may have other cellular roles. Treacle has been identified as a responder to oxidative stress in lung epithelial cells.^11^ In addition, *Tcof1* haploinsufficiency results in oxidative stress-induced neuroepithelial cell death in association with DNA damage.^12, 13^ This finding led to the hypothesis that *Tcof1* is required for protection of the neuroepithelium from oxidative stress-induced cell death.^13^

Previously, our laboratory reported the identification and cloning of the zebrafish *TCOF1* ortholog. Zebrafish *tcof1* (formerly *nolc1*-like) expression was restricted to the anterior-most region of zebrafish developing embryos,^14^ similarly to what happens in mouse embryos.^15^ The *tcof1* knockdown by using a set of two splice morpholinos (MOs) enabled a preliminary characterization of TCS craniofacial malformations in zebrafish. Among other alterations, *tcof1* knockdown caused a change in cellular nucleic acid-binding protein (*cnbp*) expression.^14^ *Cnbp* downregulation had also been reported in mouse neuroblastoma cells with diminished Treacle.^16^ CNBP, formerly zinc-finger protein 9 (ZNF9), is a single-stranded nucleic acid-binding protein strikingly conserved among vertebrates.^17, 18^ Mice, chicken and zebrafish embryos deficient in Cnbp display severely affected rostral head structures.^19, 20, 21^

The remarkable resemblance in *tcof1* and *cnbp* territorial expression and mutant phenotypes, as well as the apparent consequences of Treacle depletion on *cnbp* expression,^14, 16^ led us to consider a link between these two genes in the pathology of TCS. Here we show that *CNBP* and *TCOF1* exhibit a positive statistically significant correlation when their expression is analyzed in healthy and TCS human mesenchymal cells under chondrogenic differentiation. Furthermore, we show that Treacle depletion in zebrafish led to a lowering in the Cnbp abundance. The relevance of Cnbp in TCS is further highlighted by the finding that *cnbp* overexpression prevented craniofacial anomalies in a dose-dependent manner. The mechanism by which Cnbp mediated phenotype recovery seems to be related to a pathway that does not normalize the rRNA biosynthesis but precludes redox-responsive genes' upregulation. To our knowledge, these results are the first to make such a link not only contributing to get insight into the molecular bases of the TCS but also opening a new gate to novel treatment approaches.

## Results

### TCS modeled in zebrafish: craniofacial phenotype and molecular features

In our previous work,^14^ we used a set of two splice MOs to knockdown the expression of *tcof1* in zebrafish. Morphants generated by this experimental approach were extremely affected thus preventing detailed studies. In this work, we used an MO that by blocking the translation *tcof1*-mRNA (ATG-targeting MO (TRA-MO)) when injected in zebrafish embryos led to milder but clear and reproducible TCS-like phenotypes. The efficiency of such MO was assessed by co-injecting it with a synthetic mRNA containing the TRA-binding site upstream the enhanced green fluorescent protein (eGFP) coding sequence (Supplementary Figures S1A–C).

**Figure 1:**
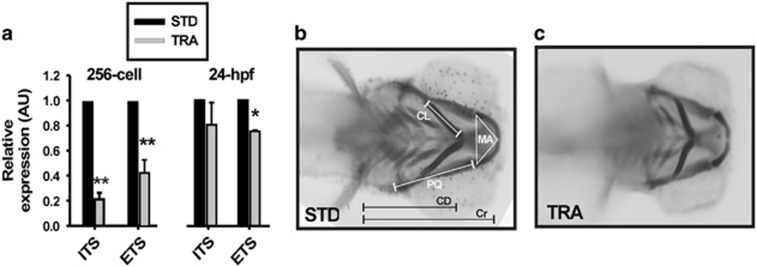
*Tcof1* knockdown in zebrafish recapitulates TCS features. (**a**) RT-qPCR of rRNA transcripts (by ETS and ITS quantification) in samples from control (STD) and TRA-MO-injected 256 cell stage and 24 hpf embryos. Bars represent the mean of relative abundance and S.E.M., *n*=3, *P<0.05, **P<0.01 (*t*-test). AU: arbitrary units. (**b** and **c**) Ventral views of a typical control (**b**; STD-MO-injected) and a representative TCS-like (**c**; TRA-MO-injected) 4 dpf larvae stained with Alcian Blue. Meckel area (MA), area of the inner triangle defined by the Meckel cartilage, PQ length: length of palatoquadrate+hyosymplectic cartilages, ceratohyal length (CL): length of ceratohyal cartilage, CD: distance between ceratohyal cartilages joint and lateral fins, cranial distance (Cr): distance between the most anterior Meckel and lateral fins

The current hypothesis states that TCS phenotypes are due to an aberrant ribosome biogenesis within neuroepithelial cells, thus enhancing apoptosis mediated by Tp53-stabilization.^10, 15^ Ribosomal DNA transcription is one of the limiting steps of ribosome biogenesis.^22^ Thereby, we explored this issue by directly measuring by real-time quantitative PCR (RT-qPCR) of the 47*S* rRNA abundance in 256-cell stage and 24 hours postfertilization (hpf) STD- and TRA-morphants. The abundance of the regions external transcribed sequence (ETS) and internal transcribed sequence (ITS) of the unprocessed transcript were reduced in TRA-morphants (at both stages) when comparing with controls (Figure 1a). This reduction reached statistical significance at the 256-cell stage for both ETS and ITS abundance and only for ETS at 24 hpf (probably owing to the increasing rate of synthesis typical of this developmental stage). To our knowledge, this is the first work showing a reduction in 47*S* synthesis as a consequence of Treacle depletion in a whole living organism.

**Figure 2:**
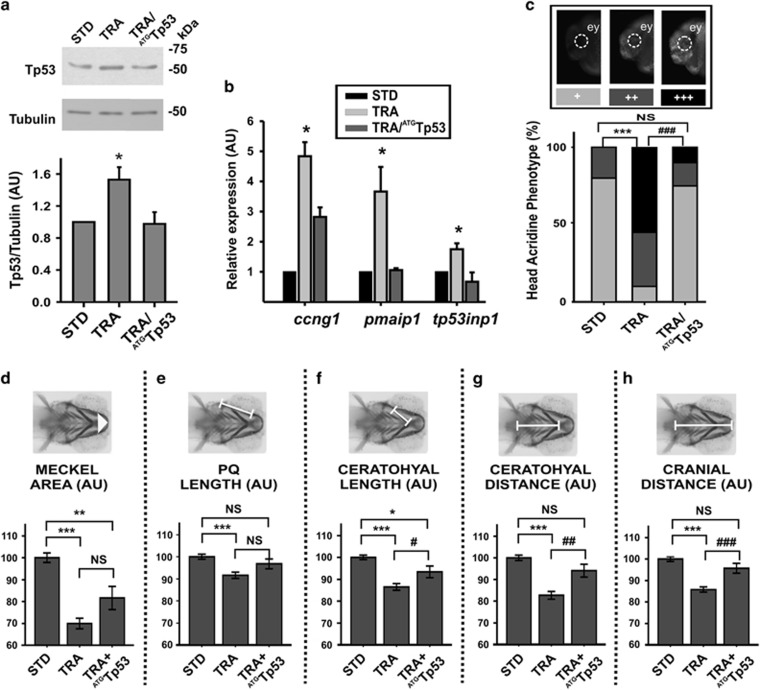
Craniofacial phenotypes generated by *tcof1* knockdown were partially rescued by Tp53 depletion. (**a**) Western blotting showing the Tp53 abundance in control (STD), TRA and TRA+^ATG^Tp53 24 hpf embryos. Molecular weight markers are shown at the right. Tubulin was analyzed as a loading control. The graph shows the densitometric quantification of western blotting band (normalization of Tp53 signal with respect to tubulin). Bars represent mean of relative abundance and S.E.M., *n*=3, *P<0.05 (*t*-test) AU: arbitrary units. (**b**) Expression analysis of Tp53 targets: *ccng1* (encoding cyclin G1), *pmaip1* (encoding Noxa) and *tp53inp1* (encoding tumor protein p53-inducible nuclear protein 1) in control (STD), TRA and TRA+ATGTp53 24 hpf embryos. Bars represent mean of relative abundance and S.E.M., *n*=3, *P<0.05, (ANOVA test). (**c**) Acridine Orange staining *in vivo* performed in 24 hpf embryos. Representative photos of embryos displaying normal (+), intermediate (++) or extensive (+++) cell death. The bar graph shows the distribution of each phenotype among the groups (STD, TRA and TRA+^ATG^Tp53 embryos). ey=eye, *n*=50 embryos in each of three independent experiments. ***P<0.001 *versus* STD; ^###^*P*<0.001 *versus* TRA; NS=non-significant *P*>0.05 (chi-square test). (**d**–**h**) Quantification of craniofacial parameters (as defined in Figure 1) measured in STD, TRA-MO TRA+^ATG^Tp53 larvae (4 dpf), stained with Alcian Blue. (**d**) Meckel Area, (**e**) PQ, (**f**) Ceratohyal length, (**g**) CD, (**h**) Cr. Bars represent means in AU and S.E.M. *n*=47 for STD, *n*=79 for TRA, *n*=30 for TRA+^ATG^Tp53. *P<0.05, **P<0.01, ***P<0.001 *versus* STD; ^#^*P*<0.05, ^##^*P*<0,01 ^###^*P*<0.001 *versus* TRA; NS=non-significant *P*>0.05 (*t*-test)

We stained 4 days postfertilization (dpf) larvae with Alcian Blue to determine whether there were defects in cranial skeleton as described for TCS subjects.^1, 3, 15^ Figures 1b and c show a typical control (universal Standard MO (STD-MO)-injected) and a representative TRA-MO-injected larvae ventral pattern of craniofacial cartilages. Among other alterations,^14^ we found that the triangular area delimited by the Meckel cartilage, the length of the palatoquadrate-hyosymplectic (PQ), the length of the ceratohyal cartilages, the distance between ceratohyal cartilages joint and lateral fins and the length of the head (estimated by the distance from the most anterior Meckel to the point where the lateral fins start) showed, compared with controls, statistically significant reductions in TRA-morphants (see below and Figures 2d–h). These types of measurements have been used by us^23^ and others^24^ to describe craniofacial phenotypes affecting cartilages. The phenotype described here is exhibited by the mutant (*nolc1*^*hi4050Tg*^, http://zfin.org/action/genotype/view/ZDB-GENO-050809-6) and is in agreement with the phenotypes and findings described in both the human syndrome and in the *Tcof1*^+/−^ mouse model.^10, 15^

To assess the role of Tp53, we co-injected in zebrafish embryos both TRA-MO and an ^ATG^Tp53 MO previously used in zebrafish.^25^ In a similar manner to that in *Tcof1*^+/−^ mice,^10^ *tp53* mRNA abundance did not statistically significantly change in Treacle-depleted zebrafish (Supplementary Fig. S1D) and, as reported for other ribosomopathies,^22^ western blotting analyses showed a Tp53 stabilization in TRA-morphants, which was normalized by the presence of ^ATG^Tp53 MO (Figure 2a). Tp53-responsive genes *ccng1*, *pmaip1* and *tp53inp1* (codifying for cyclin G1, phorbol-12-myristate-13-acetate-induced protein 1 a.k.a. Noxa and tumor protein p53-inducible nuclear protein 1, respectively) raised their expression in fish depleted of Treacle and returned to their basal levels when the translation of *tp53-*mRNA was blocked (Figure 2b). The *in vivo* observation of cells dying revealed that 24 hpf TRA-morphants displayed statistically significantly higher levels of apoptosis in cephalic region than controls. In addition, most TRA+^ATG^Tp53-morphants showed a cell death pattern similar to that detected in controls, although some still exhibited extensive cell death (Figure 2c). Finally, all the measurements performed of the craniofacial cartilages (as described above) showed an improvement when ^ATG^Tp53 was co-injected with TRA-MO in embryos (see Figure 2 legend for details and statistics). Remarkably, both the ceratohyal and the cranial distances (CD and Cr, respectively) were fully rescued by the *tp53* knockdown (Figures 2g and h).

**Figure 3:**
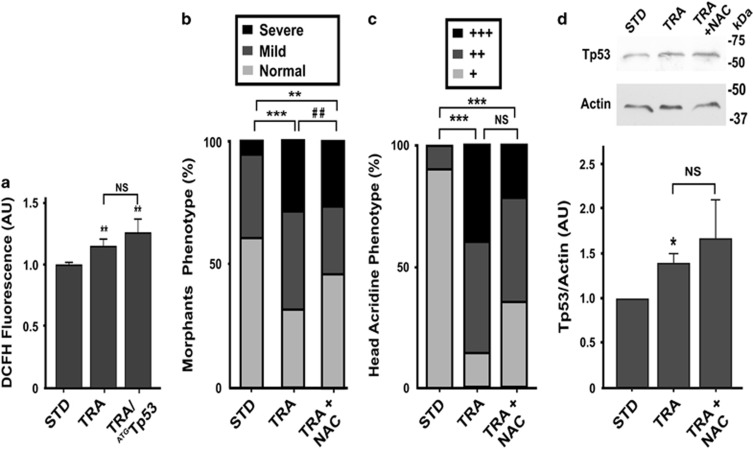
High levels of ROS in TCS embryos and prevention of craniofacial malformation through the pharmacological suppression of ROS. (**a**) Level of ROS measured in control (STD), TRA and TRA+^ATG^Tp53 48 hpf embryos. Values are expressed as the mean±S.E.M. (*n*=42). **P<0.01 *versus* STD; NS= non-significant *P*>0.05 (*t*-test) AU: arbitrary units. (**b**) Frequencies of Normal, Mild and Severe phenotypes (see Results section) in controls (STD, *n*=51), TRA (*n*=79) and TRA-larvae incubated in the presence of NAC (TRA+NAC, *n*=52). **P<0.01 ***P<0.001 *versus* STD; ^##^*P*<0.01 *versus* TRA (chi-square test). (**c**) Acridine Orange staining *in vivo* performed in 24 hpf embryos (see Figure 2c for representative photos of normal (+), intermediate (++) or extensive (+++) cephalic cell death). The bar graph shows the distribution of each phenotype among the groups (STD, TRA and TRA+NAC embryos). *n*=25 embryos. ***P<0.001 *versus* STD; NS=non-significant *P*>0.05 (chi-square test). (**d**) Western blotting showing the Tp53 abundance in STD, TRA and TRA+NAC 24 hpf embryos. Molecular weight markers are shown at the right. Actin was analyzed as a loading control. The graph shows the densitometric quantification of western blotting band (normalization of Tp53 signal with respect to Actin). Bars represent mean of relative abundance and S.E.M., *n*=3, *P<0.05 NS,non-significant *P*>0.05 (Mann–Whitney test)

Recently, it was reported that neuroepithelial cells require Treacle to deal with intrinsically amplified levels of reactive oxygen species (ROS).^13^ The assessment of the production of ROS displayed by TRA-morphants showed statistically significantly higher levels in 48 hpf TRA-morphants than in controls injected with STD-MO, and ROS levels were still high in embryos injected with both TRA-MO and ^ATG^Tp53-MO (Figure 3a). The presence of the antioxidant *N*-acetyl cysteine (NAC) in the fish media ameliorated the abnormalities of craniofacial cartilages in Treacle-depleted 4 dpf zebrafish larvae. We classified larvae as Normal when no noticeable craniofacial anomalies were observed, Mild when 1–3 phenotypic parameters (defined as in Figure 1) were affected and Severe when 4 or 5 parameters were altered (Figure 3b). The analysis of cell death by AO staining of TRA-morphants treated with NAC revealed a trend to a decrease in cell death when compared with TRA-morphants; however, differences were not statistically significant in our experimental conditions (Figure 3c). These data are in agreement with the ones recently reported by Sakai *et al.*^13^ Also, when Tp53 was analyzed in these embryos by western blotting, its signal was as high as in TRA-morphants (Figure 3d). Altogether, data presented here not only validated the TCS-like zebrafish model but also reinforced the notion of a role of ROS in the pathogenesis of TCS and suggested that ROS pathway may not be associated with Tp53 stabilization.

### Cnbp overexpression ameliorates craniofacial abnormalities in a dose-dependent manner

**Figure 4:**
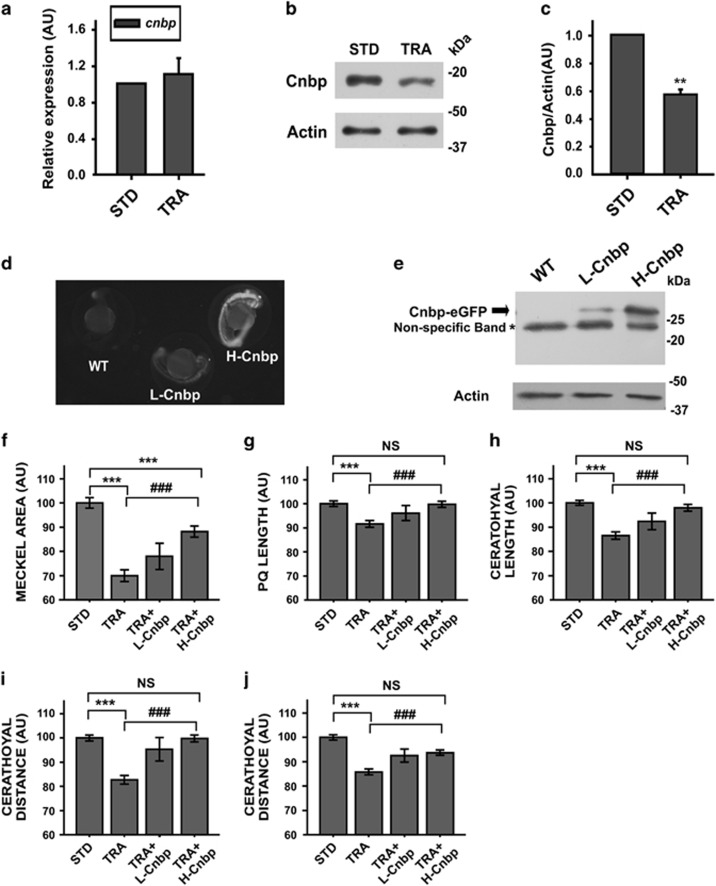
TCS-like phenotype is ameliorated in *cnbp*-overexpressing transgenic zebrafish. (**a**) Relative abundance of *cnbp* mRNA determined by RT-qPCR using total RNA from 24 hpf zebrafish control (STD) and TRA-embryos. Bars represent mean of relative abundances and S.E.M., *n*=3 AU: arbitrary units. (**b**) Western blotting analysing *cnbp* expression in 24 hpf embryos microinjected with TRA-MO. Controls were injected with STD-MO. Actin detection was used as a loading control. (**c**) Densitometric analysis of Cnbp normalized to Actin. The bars are means±S.E.M. of three experiments. *n*=5, **P<0.01 (*t*-test) (**d**) Fluorescence images of wild-type (WT) and transgenic zebrafish Tg(XIa.Eefiai:cnbpa-EGFP) lines (L-Cnbp and H-Cnbp). (**e**) Levels of Cnbp-eGFP detected by western blotting in 24 hpf transgenic zebrafish embryos by using anti-GFP antibodies. (*) Non-specific bands. Actin was assessed as a loading control. (**f**–**j**) Bar charts representing the quantification of craniofacial parameters measured in controls (STD), wild-type *tcof1*-knockdown larvae (TRA) and transgenic *tcof1*-knockdown larvae overexpressing low (TRA+L-Cnbp) or high (TRA+H-Cnbp) levels of Cnbp. (**f**) Meckel Area, (**g**) PQ length, (**h**) Ceratohyal length; (**i**) CD, (**j**) Cr. In TRA+L-Cnbp larvae, only the CD was restored (*P*<0.05 *versus* TRA and not different from STD), the Meckel area was indistinguishable from TRA and the rest of parameters showed in-between statistics (not displayed). Bars represent means in AU and S.E.M. *n*=47 for STD, *n*=79 for TRA, *n*=15 for TRA+L-Cnbp, *n*=36 for TRA+H-Cnbp. ***P<0.001 *versus* STD, ^###^*P*<0.001 *versus* TRA NS=non-significant *P*>0.05 (*t*-test)

Next we studied *cnbp* expression in our TCS-like zebrafish model. Depletion of Treacle did not affect the abundance of *cnbp* mRNA measured by RT-qPCR in embryos staged at 24 hpf (Figure 4a). However, western blotting detecting Cnbp in 24 hpf embryos injected with TRA-MO revealed a decrease in the abundance of the protein (around 50%, Figures 4b and c). To go deeper into the study of the role of Cnbp in the pathogenesis of TCS, transgenic zebrafish lines overexpressing either low level (L-Cnbp) or high level (H-Cnbp) of *cnbp* fused in frame to eGFP (Figures 4d and e) were employed. STD-MO- and TRA-MO-injected transgenic embryos were grown up to 4 dpf and cartilages' development was assessed. The area of the Meckel cartilage seemed to be restored in H-Cnbp embryos injected with TRA-MO (TRA+H-Cnbp); however, differences were still statistically significantly different when compared with control values (Figure 4f). Conversely, TRA+H-Cnbp showed full recovery of the PQ length, ceratohyal length, CD and Cr (Figures 4g–j). Compared with controls, L-Cnbp embryos injected with TRA-MO (TRA+L-Cnbp) displayed statistically significant changes in all the assessed parameters. Nevertheless, the values measured were between those ones obtained in TRA-morphants and TRA+H-Cnbp. These results suggest a dose-dependent role for Cnbp in the pathogenesis of TCS.

### Higher levels of Cnbp did not recover 47S transcription nor reverse Tp53-responsive gene expression in TCS-like zebrafish

In order to delineate a mechanism by which Cnbp abundance ameliorates craniofacial phenotypes, we analyzed whether the Cnbp increase restored 47*S* rRNA biosynthesis to control values (Supplementary Figure S2A) and/or normalized Tp53 levels, Tp53-responsive gene expression and, consequently, cell death (Supplementary Figures S2B–E). None of these parameters showed restoration to control values. Considering these results, we checked Cnbp expression in TRA+^ATG^TP53 morphant samples. Although *cnbp* mRNA was induced (Supplementary Figure S2E), Cnbp protein levels were similar to those found in TRA-morphants (Supplementary Figures S2F and G). Altogether, these results suggest that Cnbp rescue of *Tcof1* depletion-induced defects is not related to rRNA restoration or Tp53-response amelioration.

### Cnbp prevents the upregulation of redox-responsive genes in TCS-like embryos

**Figure 5:**
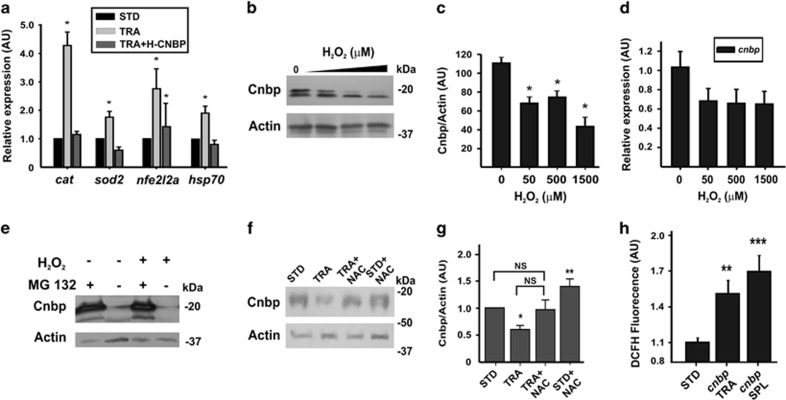
*Cnbp* overexpression prevents the upregulation of oxidative stress-responsive genes. (**a**) Expression of stress-responsive genes: *cat* (encoding catalase); *sod2* (encoding Mn-Superoxide Dismutase); *nfe2l2a* (enconding nuclear factor (erythroid-derived 2) like 2a) and *hsp70* (encoding heat shock protein 70) in controls (STD), TRA and TRA+H-Cnbp embryos staged at 24 hpf (RT-qPCR). Bars represent mean of relative abundance±S.E.M., *n*=3, *P<0.05 *versus* STD, (*t-*test). (**b**) Western blotting detection of Cnbp in 24 hpf embryos exposed to different concentrations of H_2_O_2_. Actin was assessed as a loading control. Molecular weight markers are shown at the right. (**c**) Densitometric analysis of Cnbp normalized to Actin. The bars are means±S.E.M. of three experiments, *P<0.05 (ANOVA test). (**d**) Relative abundance of *cnbp* mRNA in samples from 24 hpf embryos exposed to different concentrations of H_2_O_2_, *n*=3. (**e**) Embryos at 30% epiboly were incubated in H_2_O_2_ or control conditions in the presence or the absence of the proteasome inhibitor MG-132. At 24 hpf, the abundance of Cnbp was assessed by western blotting. Actin is the loading control. Molecular weight markers are indicated to the right. (**f**) Western blotting showing the Cnbp abundance in control (STD), TRA, TRA+NAC and STD+NAC 24 hpf embryos. Molecular weight markers are shown at the right. Actin was analyzed as a loading control. (**g**) Densitometric quantification of western blotting bands (normalization of Cnbp signal with respect to Actin). Bars represent mean of relative abundance and S.E.M., *n*=3, *P<0.05; NS=non-significant *P*>0.05 (Mann–Whitney Test) (**h**) ROS accumulation in *cnbp-*knockdown 48 hpf embryos (*cnbp* TRA and *cnbp* SPL are embryos injected with Cnbp translation or splicing MO, respectively). Each value is expressed as the mean±S.E.M. (*n*=42). **P<0.01, ***P<0.001 (*t*-test). AU: arbitrary units

Our studies disclosed that Treacle depletion causes an imbalanced redox state (Figure 3a) and a decrease in the abundance of Cnbp (Figures 4b and c). To address whether supplementation of Cnbp provides an avenue for protection against oxidative stress, we measured by RT-qPCR the expression of the two typical antioxidant enzymes catalase (*cat*) and superoxide dismutase (*sod2*), one of the major redox-sensitive transcription factors *nfe2l2a* (nuclear factor (erythroid-derived 2)-like 2) and *hsp70*, a general stress-induced gene (codifying for heat shock protein 70) in 24 hpf TRA-morphants, TRA+H-Cnbp and STD-MO-injected embryos. We observed a statistically significantly higher expression in TRA-morphantswhen comparing with STD-MO-injected embryos (Figure 5a). With the exception of *nfe2l2a*, the transcriptional expression of the analyzed genes was statistically significantly lower in TRA+H-Cnbp when comparing with TRA-morphants and similar to that measured in STD-MO injected embryos (Figure 5a). A direct effect of Cnbp on the expression of the assessed genes was ruled out because statistically significant differences between STD-MO-injected wild type and STD-MO-injected H-Cnbp embryos were not detected (not shown). These results suggest that higher levels of Cnbp ameliorate the TCS phenotypes by partially preventing the onset of the antioxidant-response process.

### The biochemical features of Cnbp may explain its role in the amelioration of craniofacial TCS-like phenotypes

The ROS sensibility of a protein is determined by the susceptibility of cysteine residues to oxidation, which is determined by the p*K*_a_ of their thiols. The microenvironment of a cysteine residue defines its reactivity.^26^ Cnbp contains 10 times more cysteine residues than the average Swiss Prot Data Bank protein (Supplementary Fig. S3A). At least three of these cysteines (see Supplementary Figure S3B) are bounded by aromatic/basic residues; thereby, we tested *in vivo* the Cnbp-ROS sensibility. Wild-type zebrafish embryos were incubated in the presence of increasing amount of H_2_O_2_ in the fish media and Cnbp protein levels were measured. A statistically significant decrease in the abundance of Cnbp was detected (Figures 5b and c) without statistically significant changes in the abundance of *cnbp* transcripts (Figure 5d). Most of ROS-sensitive proteins are degraded through the proteasome pathway.^26^ In agreement, the incubation of embryos with the proteasome inhibitor MG-132 abrogated Cnbp degradation, even in the presence of H_2_O_2_ (Figure 5e). Moreover, treatment of TRA-morphants with NAC prevented Cnbp degradation (Figures 5f and g). These biochemical features suggest that Cnbp functions as a ROS-cytoprotective protein. The potential protective role of Cnbp against ROS was strengthened by the finding that Cnbp depletion either by splice^20^ or translation-blocking^21^ MOs caused a statistically significant increase of ROS (Figure 5h). Altogether, these findings suggest that higher levels of Cnbp ameliorate the TCS phenotypes by enhancing the capability of CNCC to cope with ROS.

### CNBP expression in human control and TCS mesenchymal cells

**Figure 6:**
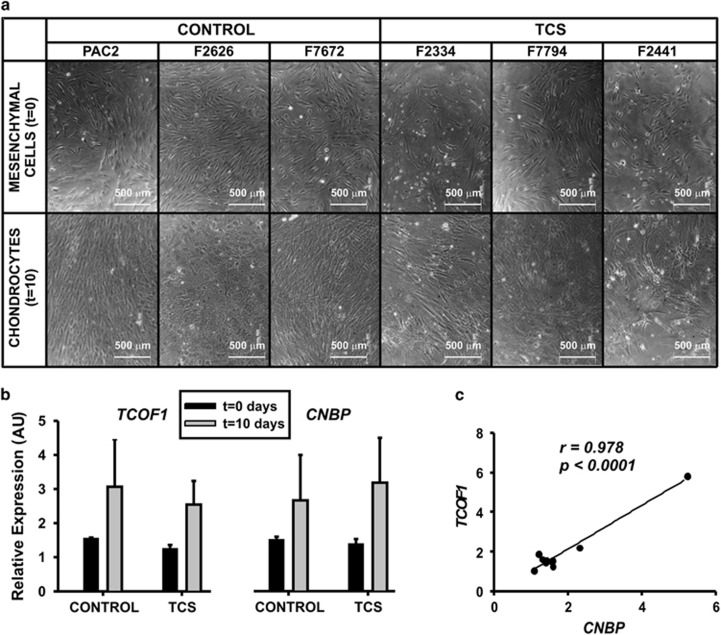
*TCOF1* and *CNBP* mRNA expression analysis in human mesenchymal cells under chondrocyte differentiation. (**a**) Bright field microscope pictures of mesenchymal cells before and after chondrogenic differentiation. Cells were obtained from healthy (control) individuals (PAC2; F2626; F7672) and TCS subjects (F2334; F7794; F2441); (**b**) Relative expression of *TCOF1* (left) and *CNBP* (right) estimated by RT-qPCR before (*t*=0 days) and after cell differentiation (*t*=10 days) in the above mentioned control and TCS cells; (**c**) Graphical representation of the correlation analysis between *TCOF1* and *CNBP.* Pearson correlation Coefficient, *r*=0.978, *P*<0.0001

*Cnbp* knockdown results in craniofacial aberrant phenotypes^21^ resembling the ones typically observed in TCS (see Supplementary Table S1). In this work, we detected decreased abundance of Cnbp in TCS-like zebrafish and phenotype amelioration owing to Cnbp overexpression. These findings led us to speculate about a role for *CNBP* in the pathogenesis of TCS. To explore CNBP role in TCS, we evaluated the expression of *TCOF1* and *CNBP* in human cells obtained from healthy (controls) and TCS subjects. TCS cells did not display differences in proliferative profile when comparing to controls (unpublished results). During the differentiation process toward chondrocytes, cells from TCS subjects failed to achieve a homogeneous distribution, and the presence of cell clusters was evident (Figure 6a). In contrast, cells from healthy individuals developed a regular monolayer pattern, with uniform appearance throughout the plate (Figure 6a). *TCOF1* and *CNBP* mRNA levels were measured in human mesenchymal cells before (day 0) and after differentiation toward chondrocytes (tenth day) (Figure 6b). There were no significant differences in the expression of *TCOF1* and *CNBP* in control or TCS cells, either under proliferation or differentiation-inducing culture conditions. However, a significant correlation between the expression of the two genes could be established by Pearson analysis (Pearson coefficient *r* was 0.978, *P*<0.0001; Figure 6c), suggesting a shared transcriptional regulation in human mesenchymal cells. This behavior was independent of *TCOF1* mutations and it was preserved during differentiation of mesenchymal cells to chondrocytes.

## Discussion

So far, most publications agreed that, in roughly 90% of the cases, the pathogenesis of TCS arises from compromised ribosome biogenesis as a direct result of *TCOF1* haploinsufficiency.^3, 4, 10, 15^ However, both the pathogenesis of TCS and the reasons of the high inter- and intra-familial variability in phenotypic severity remain unclear.^27, 28, 29^

In this work, we report a TCS-like zebrafish model that can be easily implemented and fully recapitulates the spectrum of biochemical and developmental features observed in the human syndrome^28^ and the mouse knockout model.^10, 13, 15^ Zebrafish, because of its favorable attributes such as tiny size, transparent embryo and rapid external embryonic development, has gained a preferable status among all other available experimental animal models. Using this model, decreased rRNA transcription was measured by RT-qPCR, avoiding indirect determination by immunocytochemical approaches that require complex image analysis to generate quantitative data.

As reported in other ribosomopathies,^30, 31^ we found an increased accumulation of ROS in *tcof1*-knockdown zebrafish. The relative increase of ROS in addition to the upregulation of antioxidant-response genes suggests a diminished capability of *tcof1*-knocked down embryos to properly manage the cellular redox balance. In this sense, Sakai *et al.*^13^ have recently reported that neuroepithelial cells exist endogenously in a highly oxidative state and, in addition to its role in ribosome biogenesis, Treacle is required for protection of the neuroepithelium from oxidative stress-induced cell death. This finding led to suggest that the variability in phenotypic severity is a consequence of varying levels of oxidative stress depending on the physiological conditions in the mother.^13^ The results presented in this work strengthen the notion about the involvement of oxidative stress in the pathogenesis of TCS and the possibility of preventing it by antioxidant supplementation during development.

The partial reversion of TCS-like craniofacial phenotypes in the Tp53-knockdown zebrafish embryos might be due to the presence of Tp53 isoforms in zebrafish.^32, 33^ In agreement, Lau *et al.*^34^ have recently reported a partial rescue of the facial POLR1C-dependent Type 3 TCS phenotype in the background of *Tp53* mutants. Altogether, it appears that increased CNCC apoptosis in TCS-like zebrafish is due to Tp53 stabilization along with other altered cellular processes, such as misbalanced redox homeostasis^35^ and/or distorted Cnbp turnover.

In the case of maternally inherited genes, such as *tcof1*, the knockdown by translation MO usually generates phenotypes more severe than those generated by MO affecting the pre-mRNA processing. However, herein the use of TRA-MO generated a milder condition, probably because the splicing MO also generated RNAs encoding Treacle dominant-negative isoforms.^14^ This mild condition may explain the absence of significant differences not only in the relative abundance of *cnbp* transcripts but also in the expression of its targets, *tbx2b* and *tcf7l2*.^23, 36^ Nevertheless, the strong statistical correlation between *CNBP* and *TCOF1* expression in human mesenchymal cells further supports the hypothesis of a functional link between these two genes in the pathology of TCS. This hypothesis is strengthened by the finding that increased expression of *cnbp* rescued the aberrant craniofacial phenotype of *TCS*-like zebrafish in a dose-dependent manner.

**Figure 7:**
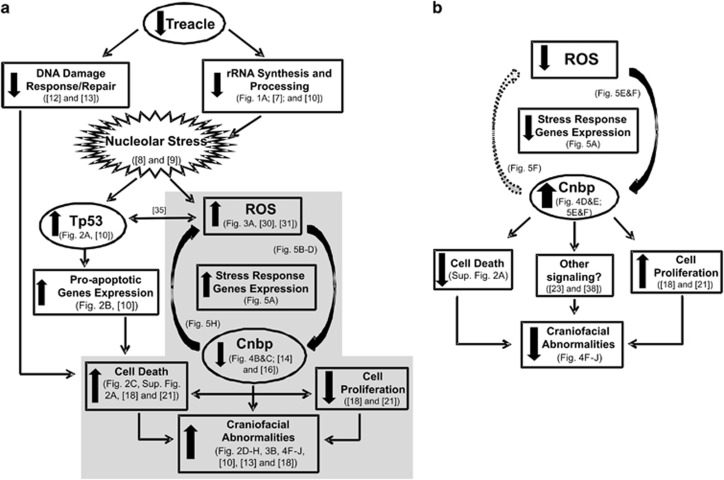
Schematic representation of the proposed TCS pathogenesis model. (**a**) *Tcof1* haploinsufficiency leads to deficient rRNA synthesis and processing and consequent nucleolar stress. Nucleolar disruption results in: (i) stabilization of Tp53 and upregulation of pro-apoptotic genes; and (ii) an increase of cellular ROS, with the resultant upregulation of stress-response genes and decreased abundance of Cnbp. In addition to its role in ribosome biogenesis, Treacle may also be required in DNA damage repair response for protection of the neuroepithelium from oxidative stress-induced cell death. These events lead to an increase of cell death and decrease of cell proliferation, which result in facial abnormalities. Shaded in grey: ROS and Cnbp in TCS pathogenesis. (**b**) Proposed mechanism for craniofacial defects rescued by Cnbp. Overexpression of *cnbp* in TCS-like fish prevents the redox-responsive genes' upregulation and leads to decrease of cell death and craniofacial malformations

Cnbp binds to G-rich sequences of DNA capable of folding as G-quadruplex non-canonical secondary structures,^36^ and rDNA transcription is partially controlled by G-quadruplexes.^37^ So, our first hypothesis was that Cnbp was improving rRNA biosynthesis. However, this hypothesis was ruled out by RT-qPCR data showing no variations in the abundance of the 47*S* in TCS-like embryos overexpressing *cnbp*. In a similar manner, Tp53 and its responsive genes were induced even in Treacle-depleted embryos overexpressing Cnbp, suggesting that Cnbp acts independently of Tp53 for rescuing TCS-like phenotype. A more careful analysis of Cnbp primary structure highlighted a putative sensibility to ROS, which was verified by *in vivo* exposing embryos to H_2_O_2_ and checking Cnbp degradation through the proteasome pathway (emblematic of ROS-sensitive proteins^26^ and recently reported for Cnbp in mammalian cells^38^). Moreover, Cnbp degradation was prevented by NAC treatment in embryos injected with TRA or even STD MOs. The lowering in Cnbp abundance in TCS-like zebrafish was concurrent with the upregulation of redox-responsive genes and an accumulation of ROS. In addition, the comparison of laboratory strains of zebrafish expressing different amount of Cnbp (L-Cnbp and H-Cnbp fish lines) revealed differential susceptibility to Treacle depletion. Based on this, we speculate that higher levels of Cnbp could raise the threshold for oxidative stress, such that CNCC might be less sensitive to ROS in a context of Treacle deficiency. Altogether, these findings suggest a potential ROS-cytoprotective role for Cnbp during embryonic development. Figure 7 shows our working model of TCS pathogenic mechanism and depicts the possible cytoprotective role of Cnbp. In this connection, Cnbp degradation inhibition (by ROS scavengers or proteasome inhibitors) can be considered as a potential preventive therapy for TCS.

The progress in DNA sequencing technology and genome-wide screen approaches have led to an increased awareness that the genetic background of an organism strongly influences the phenotypic outcome under a given environmental impact. Thus it is tempting to speculate that the clinical variability of TCS is—in some measure—due to variations of the genetic background affecting the Cnbp expression. In this context, the existence in mice of distant-acting *cnbp* enhancers likely contributing to the fine-tuning of craniofacial morphology was recently reported.^39^

In conclusion, the results presented here suggest that the pathogenesis of TCS includes novel mechanisms involving Cnbp as a ROS-cytoprotective protein. Moreover, these data increases our understanding about the pathogenesis of TCS and suggest CNBP as an additional target for new alternative therapeutic treatments to reduce craniofacial defects not only in TCS but also in other neurocristopathies.

## Materials and Methods

### Materials

Otherwise indicated, all chemicals were purchased from Sigma-Aldrich (Saint Louis, MO, USA).

### Animal handling and ethics statement

This study was carried out in strict accordance with relevant national and international guidelines. Protocols were approved by the Committee on the Ethics of Animal Experiments of the Facultad de Cs. Bioquímicas y Farmacéuticas-UNR (http://www.saludinvestiga.org.ar/comites.asp?num_prov=13). Adult zebrafish were maintained at 28 °C on a 14 h light/10 h dark cycle. Embryos were staged according to development in hpf at 28 °C.^40^ An inbred strain was used as wild type. Heterozygous Tg (XIa.Eefiai:cnbpa-EGFP) fish overexpressing *cnbp* fused to eGFP were also employed (http://www.zfin.org).

### Microinjection of zebrafish embryos and chemical treatments

Embryos were injected at ≤4 cell stage using a gas-driven microinjection apparatus (MPPI-2 Pressure Injector, Applied Scientific Instrumentation; Eugene, OR, USA). To repress *tcof1* mRNA translation, TRA-MO (5′-TAGGAACCGTGCTGTCCTCCGCCAT-3′) was used at the concentration of 0.5 pmol/embryo (4 ng/embryo) in 5 nl as previously reported.^21, 23^ As a control, STD-MO (5′-CCTCTTACCTCAGTTACAATTTATA-3′) was injected. ^ATG^Tp53 was used as described in Ning *et al.*^25^ Cnbp TRA and Cnbp SPL were used as described.^21, 41^ All MO were synthesized by Gene Tools LLC (Philomath, OR, USA).

TRA-MO targeting efficiency was assessed as suggested by Bill *et al.*^42^ Briefly, a 172-bp region of the zebrafish *tcof1* gene containing the TRA-MO-binding site was cloned upstream of eGFP-coding sequence (Supplementary Figure S1A). The plasmid was used for *in vitro* transcription (mMESSAGE mMACHINE Kit, Thermo Fisher Scientific, Waltham, MA, USA) following the manufacturer's instructions. Embryos were co-injected with 50 pg of mRNA and 4 ng of TRA-MO or STD-MO, and then the eGFP expression was analyzed under MVX10 Olympus Fluorescence Microscope (Tokyo, Japan). According to previous report,^43^ the off-targeting effects were ruled out because the expression of *tp53* measured by RT-qPCR did not significantly change (Supplementary Figure S1D).

Embryos were exposed to H_2_O_2_ (Cicarelli, San Lorenzo, Argentina) at 0, 50, 500 and 1500 *μ*M for 18 h (from 5 hpf to 24 hpf) to generate oxidative stress conditions. To inhibit proteasome activity, MG-132 (Santa Cruz Biotechnology, CA, USA) was added at a concentration of 5 *μ*M 1 h before H_2_O_2_ treatment. For antioxidant-rescue experiments, injected embryos were incubated in E3 containing 100 *μ*M NAC from 5 hpf to 4 dpf; solutions were changed daily.

### Zebrafish phenotypic analyses

Four dpf larvae were fixed, stained with Alcian Blue as described elsewhere^21^ and observed with a MVX10 Olympus Microscope equipped with a MVXTV1XC Olympus digital camera. Cranial cartilages measurements (see Figure 1) were determined using the ImageJ software (National Institutes of Health, Bethesda, MD, USA).^44^

Injected 24 hpf embryos were manually dechorionated and stained with the vital dye Acridine Orange (Sigma, St. Louis, MO, USA) as described.^41^

### Zebrafish molecular analysis

ROS abundance was detected in injected 48 hpf embryos by measuring the intracellular level of the oxidized form of acetyl ester of dichloro-dihydro-fluorescein diacetate, as described elsewhere.^45, 46^

For western blotting, 24 hpf embryos were dechorionated and processed according to Link *et al.*^47^ SDS-PAGE was performed essentially according to Laemmli.^48^ Gels were electrotransferred to nitrocellulose membranes according to Towbin *et al.*^49^ Rabbit polyclonal anti-actin (I-19; sc-1616-R), mouse monoclonal anti-tubulin (TU-02; sc-8035) and rabbit polyclonal anti-p53 (FL-393; sc-6243) were from Santa Cruz Biotechnology. Rabbit polyclonal anti GFP (ab290) was from Abcam (Cambridge, UK). Cnbp anti-serum was raised in rabbit against the zebrafish full-length protein.^41^ Membranes were incubated with the appropriate primary and HRP-conjugated antibodies (1/7000 to 1/25000 dilution), washed and developed using chemiluminescence (SuperSignal West Pico Chemiluminescent Substrate, Thermo Fisher Scientific) and X-ray films (Amersham Hyperfilm ECL, GE Healthcare Science, Chicago, IL, USA).

Relative protein concentrations were determined according to Sedmak and Grossberg,^50^ using bovine serum albumin as standard.

Total RNA from 24 hpf embryos was obtained using TRIzol Reagent (Invitrogen, Carlsbad, CA, USA) and following the manufacturer's instructions. Purified RNA was incubated with RQ1 DNAse (Promega, Madison, WI, USA) and oligo(dT) retrotranscribed with M-MLV reverse transcriptase (Promega). Quantification reactions were performed using three different RNA purifications from three independent microinjection experiments using an Eppendorf Realplex2 apparatus and SYBR green I (Invitrogen) chemistry. Each reaction tube (20 *μ*l) consisted of 0.5 × SYBR Green I, 0.2 *μ*M of each primer (Supplementary Table S2), 2.5 mM MgCl_2_, 0.2 mM dNTPs, 0.5 U Platinum Taq DNA polymerase (Invitrogen) and 2 *μ*l of template/negative controls. *Ef1α* and *Rlp13a* were used as endogenous controls for gene expression normalization.^51^ Relative gene expression values were calculated using qBase v 1.3.5.^52^ Statistical differences were analyzed by ANOVA and Student's *t*-tests. The validity of the RT-qPCR data was assured by following the MIQE guidelines.^53^

Ribosomal RNA transcription was estimated via RT-qPCR as elsewhere.^22^ Briefly, two pairs of primers (Supplementary Table S2) were designed against the 5′ETS and ITS1 regions of the unprocessed transcript (47*S*) based on Azuma *et al.*^54^ Total RNA from 256-cell stage and 24 hpf embryos were used in retro-transcription reactions using random primers. *Actin* and *Rlp13a* were used as endogenous controls for gene expression normalization.

All the oligonucleotides used (Supplementary Table S2) were purchased from GenBiotech (http://www.genbiotech.com.ar/). Specific oligonucleotide primers for each gene under study were designed using Primer-BLAST (http://www.ncbi.nlm.nih.gov/tools/primer-blast/) and their specificity checked using MFE http://biocompute.bmi.ac.cn/CZlab/MFEprimer-2.0/.

### Studies with mesenchymal stem cells from TCS patients and controls

We studied Stem cells from Human Exfoliated Deciduous teeth (SHED) obtained from three TCS subjects and from three control individuals subjected to reconstructive plastic surgery at the University of São Paulo Medical School (See Supplementary Table S3 for patients' details). The study was approved by the ethical committee of the Institution and informed consent was obtained from both patients and control individuals or from their legal tutors. SHED were isolated as described by previous reports.^55^ SHED were cultured and expanded in monolayer in DMEM/F12 (Gibco, Thermo Fisher Scientific) plus 15% fetal bovine serum (Gibco, Life Technologies), 1% Penicillin and Streptomycin and 1% non-essential amino acids (Gibco, Life Technologies) at 37 °C in a humidified atmosphere of 5% CO_2_. Differentiation to chondrocytes was carried out in StemPro Chondrogenesis Differentiation Kit (Gibco, Life Technologies) during 10 days.^56^ RNA samples were extracted at day 0 (before *in vitro* chondrogenesis) and at day 10 (after *in vitro* chondrogenesis) using the NucleoSpin RNA II (Macherey-Nagel, Düren, Germany) Extraction Kit. Reverse transcription was performed using Superscript II Reverse Transcriptase (Invitrogen, Life Technologies). RT-qPCR reactions were performed in triplicate using 2 × SYBR Green PCR Master Mix (Life Technologies) and an ABI Prism 7500 Sequence Detection System (Applied Biosystems, Life Technologies). Primer sequences are listed in Supplementary Table S2. GeNorm v3.4^57^ was used to determine the most stable endogenous controls (among *TBP*, *HPRT*, *GADPH* and *HMBS*) and to calculate normalization factors for each sample. The final expression values were determined based on a previous method (qBase v 1.3.5) and then analyzed statistically using the GraphPad Prism program v5.03 (http://www.graphpad.com/scientific-software/prism/).

## Abbreviations

CD: ceratohyal distance
CNBP: cellular nucleic acid-binding protein
CNCC: cranial neural crest cell
Cr: cranial distance
ETS: external transcribed sequence
ITS: internal transcribed sequence
MO: morpholino
NAC: *N*-acetyl cysteine
PQ: palatoquadrate-hyosymplectic
ROS: reactive oxygen species
STD-MO: universal standard morpholino
TCS: Treacher Collins Syndrome
TRA-MO: ATG-targeting morpholino against *tcof1*
