# Supplementary figures and images for: Cnbp ameliorates Treacher Collins Syndrome craniofacial anomalies through a pathway that involves redox-responsive genes
Source: Cell Death Dis. 2016 Oct 6;7(10):e2397–. doi: 10.1038/cddis.2016.299 (PMC5133970; doi:10.1038/cddis.2016.299)

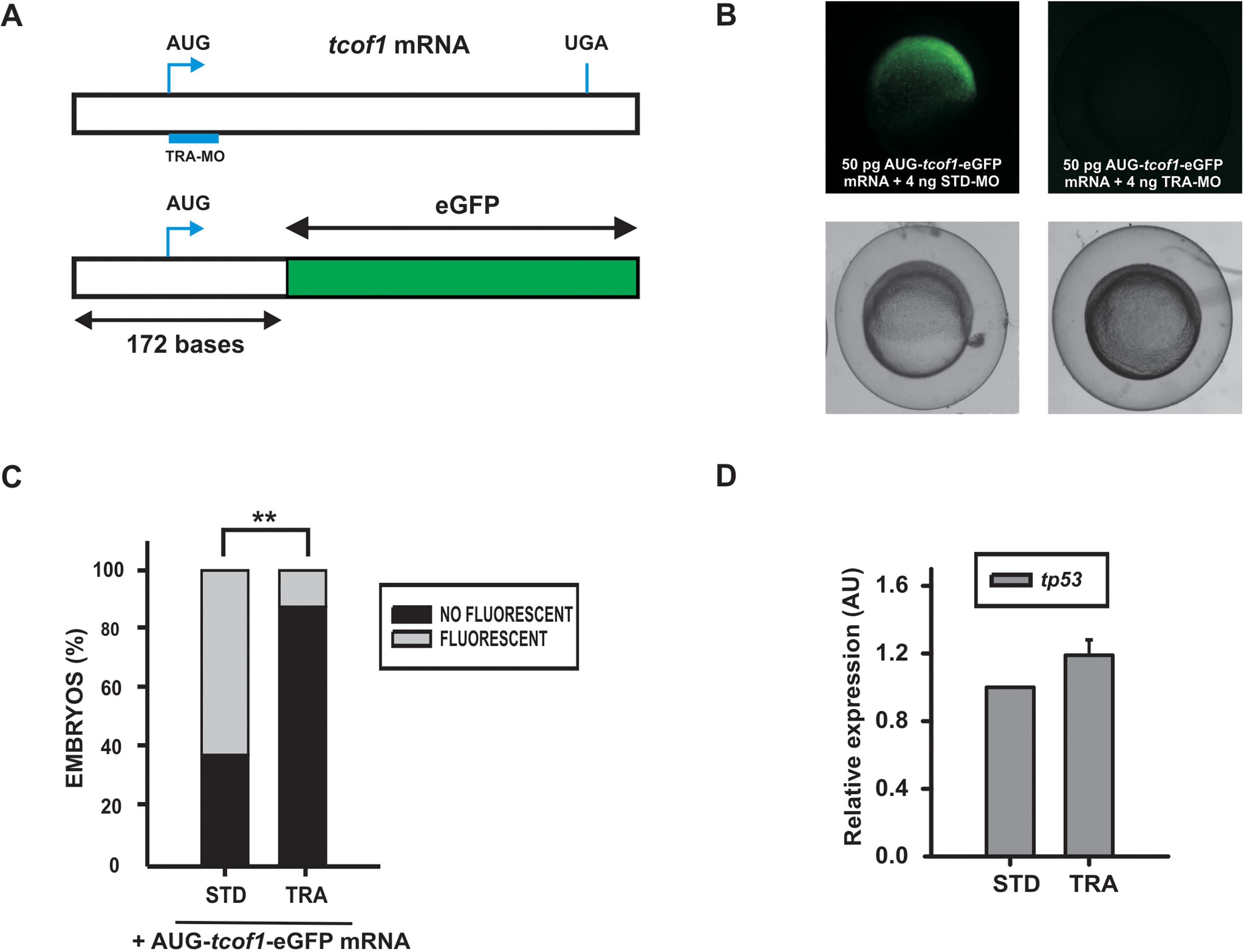

Supplement: Supplementary Figure S1 [file cddis2016299x1.tif]

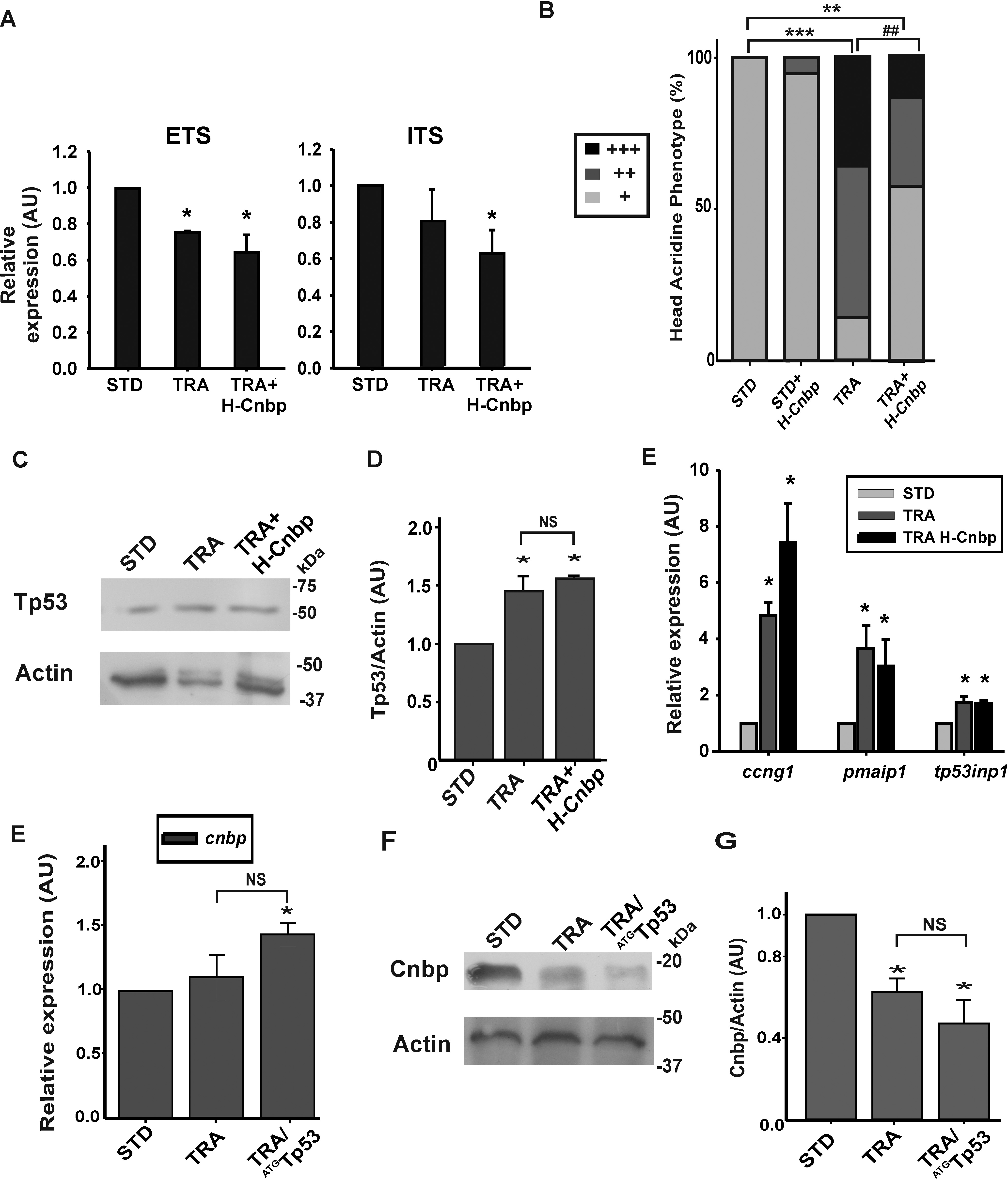

Supplement: Supplementary Figure S2 [file cddis2016299x2.tif]

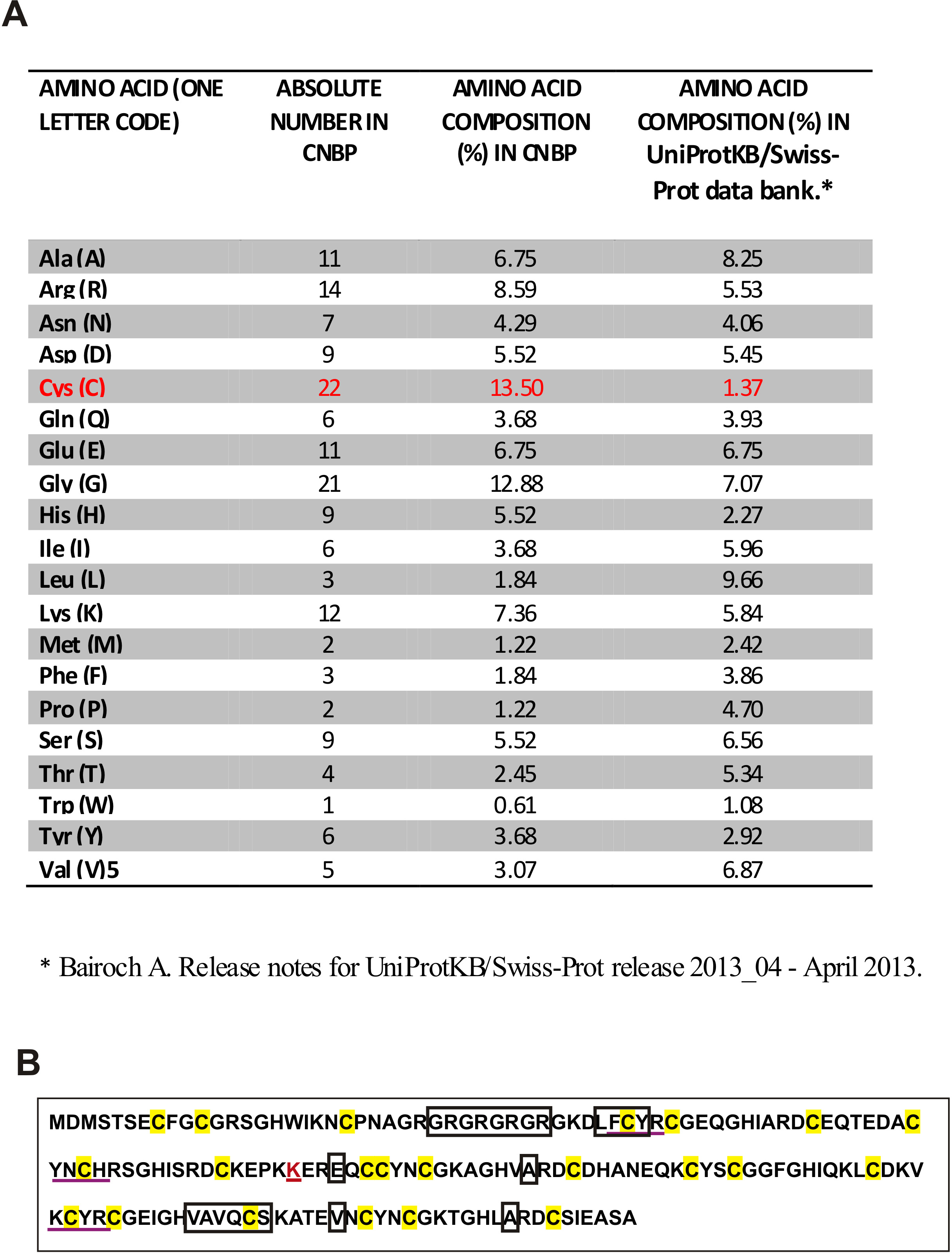

Supplement: Supplementary Figure S3 [file cddis2016299x3.tif]
