# Supplementary material for: Cnbp ameliorates Treacher Collins Syndrome craniofacial anomalies through a pathway that involves redox-responsive genes
Source: Cell Death Dis. 2016 Oct 6;7(10):e2397–. doi: 10.1038/cddis.2016.299 (PMC5133970; doi:10.1038/cddis.2016.299)
Supplement: Supplementary Tables [file cddis2016299x4.docx]

**Supplementary Table S1:** Zebrafish embryo phenotypes obtained after *cnbp* or *tcof1* knock-down.

|  | ***cnbp* depletion** | ***ztcof1* depletion** |
| --- | --- | --- |
| **Meckel´s cartilage** | Failure of anterior extension | Not extended anteriorly |
| **Palatoquadrate** | Reduced size | Reduced size |
| **Hyosymplectic** | Reduced size | Reduced size |
| **Pterygoid process,** | Reduced size | Reduced size |
| **Ceratohyal Arches** | Malformed | Malformed |
| **Angle formed by ceratohyal cartilages** | obtuse | obtuse |
| **Ceratobrachial arches 1-5** | Difficult to see | Difficult to distinguish |
| **Neurocranial cartilages**  **Anterior edge of the ethmoid plate** | Reduced size  Dysmorphic/shorter | Reduced size  Smaller and dysmorphic |
| **Chondrocytes**  **-morphology**  **-organization**  **-Size**  **-Shape**  **-Number** | Abnormal  Disordered  Larger  More round  Reduced (a half) | Abnormal  Disordered  Larger  More round |
| **Pharyngeal Arches** | Hypoplastic | Reduced |
| **References** | Weiner et al., 2007. | Weiner et al., 2012; this manuscript |

**Supplementary Table S2:** Oligonucleotides used in this study.

|  | **Sequence (5´-3´)** | | **Amplified fragment** | **Acession** |
| --- | --- | --- | --- | --- |
| **Target gene** | **Forward** | **Reverse** | **(base pairs)** | **Number** |
| *ef1a* | CTGGAGGCCAGCTCAAACAT | ATCAAGAAGAGTAGTACCGCTAGCATTAC | 87 | FJ915061.1 |
| *rpl13a* | TCTGGAGGACTGTAAGAGGTATGC | AGACGCACAATCTTGAGAGCAG | 148 | NM_212784.1 |
| *βact* | CGAGCAGGAGATGGGAACC | CAACGGAAACGCTCATTGC | 102 | AF057040.1 |
| *ccng1* | CATCTCTAAAAGAGGCTCTAGATGG | CACACAAACCAGGTCTCCAG | 71 | AY423016.1 |
| *pmaip1* | ATGGCGAAGAAAGAGCAAAC | CGCTTCCCCTCCATTTGTAT | 134 | DQ860152.1 |
| *trp53inp1* | AGGTTCCGGTCTTCCTGAATGTGT | GCAACGCAATTAAAGGCAACGAAA | 175 | NM_001080036.1 |
| *cnbp* | GCTTTGGGCACATCCAGAAAT | CCGCAGTTGTAGCAGTTCAC | 115 | NM_199749.2 |
| *ztcof1* | CCGGAATTCCCACGTGAGTGCAACGAAGATGGC | AAACTGCAGAAGATGTCCATAAGACTGTCC | 172 | NM_001256652.1 |
| *5ETS* | GCGAAGCACGTCGCATTTCC | TCCTCGCTCACGGGTCTCG | 120 |  |
| *ITS1* | TGGGTTCAAAGACCTTCCCGTC | CAGGGTCCGACACCACAAAGAG | 109 |  |
| *nfe2l2a* | TCACTCCCAGAGTTGCAGCAG | CTGTTTGAGCCGAGCCGAG | 148 | AB081314.1 |
| *hsp70* | CAAAGGCAAATCCTCAGA | CACAAAGTGGTTCACCATGC | 169 | AF210640.1 |
| *cat* | CAACAACCCTCCAGACAGACC | CGACCCTCCTTCCACAGTTTC | 143 | NM_130912.1 |
| *sod2* | CCGGACTATGTTAAGGCCATCT | ACACTCGGTTGCTCTCTTTTCTCT | 123 | AY195857.1 |
| *CNBP* | CATGCGTGTTAATGGCTGTT | TGCATTTCTCTGCAATTCCT | 207 | NM_001127192.1 |
| *TCOF1* | GCCCCTGGAAAAGTTGTCACT | GGTTTCTCACTGGTGGCTTCAC | 79 | U76366.1 |
| *TBP* | GTGACCCAGCATCACTGTTTC | GCAAACCAGAAACCCTTGCG | 138 | NM_003194.4 |
| *HPRT* | CCTGGCGTCGTGATTAGTGAT | AGACGTTCAGTCCTGTCCATAA | 131 | NM_000194.2 |
| *GADPH* | ATCACCATCTTCCAGGAGCG | GGGCAGAGATGATGACCCTTT | 151 | NM_002046.5 |
| *HMBS* | AGCTTGCTCGCATACAGACG | AGCTCCTTGGTAAACAGGCTT | 157 | NM_000190.3 |

**Supplementary Table S3:** Pathogenic mutation and main clinical features of the TCS subjects.

| TCS  Subject | *TCOF1*  Mutation | Clinical  Features |
| --- | --- | --- |
| F2334 | c.218_222 ins AACC | - Conductive deafness. - Malformation of auricles. - Midface hypoplasia. |
| F2441 | c.4344dupA | - Antimongoloid slanting palpebral fissures. - Lower lid coloboma. - Retrognatia. - Respiratory problems due to narrow airway. |
| F7794 | c.885_866 del | - Conductive deafnes. - Antimongoloid slanting palpebral fissures. - Malformation of auricles. - Retrognatia, facial asymmetry. |
